# Diversity of Drought Tolerance in the Genus *Vigna*

**DOI:** 10.3389/fpls.2018.00729

**Published:** 2018-06-15

**Authors:** Kohtaro Iseki, Yu Takahashi, Chiaki Muto, Ken Naito, Norihiko Tomooka

**Affiliations:** Genetic Resources Center, National Agriculture and Food Research Organization, Tsukuba, Japan

**Keywords:** genus *Vigna*, wild species, drought stress tolerance, genetic diversity, evolution

## Abstract

Wild relatives of crop plants are thought as reservoir of prominent genetic resources for abiotic stress tolerance. However, insufficient information on genetic variation and phenotypic traits restricts their use for crop breeding. This study focused on wild species of genus *Vigna* (family Fabaceae) originated from highly humid to arid regions. To clarify the diversity of drought tolerance during the vegetative stage, 69 accessions, including 15 domesticated, and 54 wild accessions, were evaluated under two drought conditions of non-terminal and terminal stresses. In the non-terminal drought condition, the plants were grown in pipes of different heights where surface soil water content decreased faster in pipes with greater height. Relative shoot biomass was used for tolerance evaluation and we identified 19 drought tolerant accessions. Almost of them were wild accessions showing higher relative shoot biomass than that in the domesticated accessions. Domesticated species were mostly classified as drought susceptible but could be improved using tolerant conspecific wild ancestors with cross-compatibility. The tolerance was related with higher plant water status presumably due to small water consumption. However, the variation of drought tolerance could not be explained by simple tolerance factor alone, and other tolerance mechanisms such as deep rooting and increasing in root biomass were found in the tolerant accessions. In the terminal drought condition, the plants were grown in small pots, and the watering was stopped to expose them extreme and rapid soil water scarcity. The tolerance was evaluated as the number of days until wilting. However, the accessions found to be tolerant in the pot experiment were not the same as those in the pipe experiment. In this condition, plant water status was not related with the length of days to wilting. This indicates that different mechanisms are necessary for adaptation to each of the non-terminal and terminal drought conditions. Many accessions were tolerant to one of the conditions, although we identified that some accessions showed tolerance in both experiments. The great diversity in drought tolerance in the genus *Vigna* might serve to both improve crop drought tolerance and understand the mechanisms of adaptation in drought-prone environments.

## Introduction

Wild relatives of crop plants, which are well adapted to the natural environments, represent potential genetic resources to improve abiotic stress tolerance in crops. To match the global food demand in the coming century, breeding of food and forage crops that can maintain a high yield under unfavorable conditions is the first step (Sanchez, 2015). To improve stress tolerance in crops, many studies have been conducted to identify tolerant genotypes against abiotic stresses for domesticated accessions (Munns and James, 2003; Torres et al., 2013; Sardouie-Nasab et al., 2014; Monkham et al., 2015). However, domesticated accessions typically show limited genetic variation due to the domestication bottleneck (Hyten et al., 2006; Zhu et al., 2007). This situation decreases the opportunity to breed crops having variation in environmental adaptability.

In contrast, wild relatives of crop plants often have a greater adaptability to abiotic stresses (Mittova et al., 2002; Baum et al., 2003; Koziol et al., 2012) and are expected to have valuable genes for breeding (Palmgren et al., 2014). However, a lack of genetic and phenotypic information about such wild relatives restricts their use (Mccouch et al., 2013). The lack of genetic information should be alleviated soon, as it has become easier to sequence the whole genome of even non-model plants (Sakai et al., 2015). Therefore, it is even more important to comprehensively evaluate wild crop relatives for agronomically important traits including abiotic stress tolerance.

As a gene reservoir for drought tolerance, we focused on wild species in the genus *Vigna*, which comprises nine domesticated and more than 100 wild species. The origins of wild species range from highly humid regions with annual rainfall up to 4,000 mm, to arid regions that receive <100 mm rain (Tomooka et al., 2014) indicating genetic diversity for drought tolerance. Although great genetic variations are expected in this genus, previous studies on drought tolerance of *Vigna* species have focused on domesticated accessions (Agbicodo et al., 2009; Kumar and Sharma, 2009; Jørgensen et al., 2010); therefore, the nature of drought tolerance in wild *Vigna* is not well understood. To use these wild genetic resources for crop breeding, we addressed to clarify detailed genetic variation and phenotypic information on drought tolerance.

One of the problems in drought tolerance screening is that different tolerance mechanisms are required in different growth stage or different drought conditions. Effects of drought stress on vegetative and reproductive processes would cause yield reduction in different manners (Blum, 1996; Sanchez et al., 2012). Therefore, physiological traits needed for tolerance in each growth stage is also different. In addition, tolerant mechanisms are expected to be different depending on drought conditions. This was clearly shown in a previous study by Clauw et al. (2014), where the expressed gene sets were largely different between *Arabidopsis* plants under mild and severe drought conditions. Therefore, it is important to screen plants separately in different growth stage and drought conditions to characterize drought tolerance.

In the present study, we investigated the drought stress at the vegetative stage. This is because dry spells at the vegetative stage is one of the major constraints on yield in drought-prone areas, although the effects on grain yield reduction are lesser than that caused by a drought at the reproductive stage (Daryanto et al., 2015). In addition, *Vigna* species are widely used as food crops and forage crops (Boe et al., 1991; Abd El-Salam et al., 2013); therefore, shoot biomass production under drought during the vegetative stage is an important trait also for livestock production. To evaluate genetic variation and plant traits of a diverse set of 69 *Vigna* accessions, we evaluated the responses of shoot growth under two types of drought (i.e., non-terminal drought in a pipe experiment and terminal drought in a pot experiment) during the vegetative stage. We attempted to identify tolerant accessions in each experiment and discussed plausible mechanisms. Further, we highlighted the levels of drought tolerance of the domesticated accessions and the evolutionary origins of drought tolerance in the wild accessions.

## Materials and methods

### Plant materials and drought treatments

The *Vigna* accessions used in this study are listed in Table 1. All accessions were obtained from the Genetic Resources Center, National Agriculture and Food Research Organization (NARO), Japan. We used 69 accessions, comprising 15 domesticated (nine species) and 54 wild accessions (28 species). We used the same accessions that were earlier used for salt tolerance study (Iseki et al., 2016).

**Table 1:** *Vigna* accessions used in this study.

**Subgenus/Section**	**ID**	**Species**	**Types**	**Origin**	**Accession number**
*Ceratotropis/Angurares*	1	*V. angularis* var. *nipponensis*	Wild	Japan	JP87910
	2	*V. angularis* var. *nipponensis*	Wild	Laos	JP226665
	3	*V. angularis* var. *angularis*	Domesticated	Japan	JP37752
	4	*V. tenuicaulis*	Wild	Myanmar	JP227438
	5	*V. nepalensis*	Wild	Nepal	JP107881
	6	*V. tenuicaulis*	Wild	Thailand	JP109682
	7	*V. umbellata*	Domesticated	Japan	JP99485
	8	*V. umbellata*	Wild	Thailand	JP109675
	9	*V. umbellata*	Wild	Thailand	JP210639
	10	*V. hirtella*	Wild	Thailand	JP109681
	11	*V. hirtella*	Wild	Sri Lanka	JP218935
	12	*V. exilis*	Wild	Thailand	JP205884
	13	*V. hirtella*	Wild	Thailand	JP108562
	14	*V. hirtella*	Wild	Laos	JP220137
	15	*V. reflexo-pilosa* var. *glabra*	Domesticated	Philippines	JP109684
	16	*V. reflexo-pilosa* var. *reflexo-pilosa*	Wild	Malaysia	JP108867
	17	*V. nakashimae*^†^	Wild	Japan	JP107879
	18	*V. riukiuensis*	Wild	Japan	JP108810
	19	*V. minima*	Wild	Indonesia	JP218938
	20	*V. minima*^†^	Wild	Papua New Guinea	JP226877
	21	*V. minima*	Wild	Thailand	JP107869
	22	*V. dalzelliana*	Wild	India	JP235419
	23	*V. dalzelliana*	Wild	Myanmar	JP210811
	24	*V. trinervia*	Wild	Thailand	JP108840
*Ceratotropis/Ceratotropis*	25	*V. radiata*^†^	Domesticated	Thailand	JP110830
	26	*V. radiata* var. *sublobata*	Wild	Madagascar	JP107877
	27	*V. radiata* var. *sublobata*	Wild	Papua New Guinea	JP226874
	28	*Vigna* sp. NI1135	Wild	India	JP110836
	29	*V. mungo*	Domesticated	India	JP109668
	30	*V. mungo* var. *silvestris*	Wild	India	JP107874
	31	*V. sahyadriana*	Wild	India	JP235420
	32	*V. grandiflora*^†^	Wild	Thailand	JP107862
*Ceratotropis/Aconitifoliae*	33	*V. khandalensis*	Wild	India	JP253828
	34	*V. subramaniana*	Wild	India	JP229284
	35	*V. subramaniana*	Wild	India	JP229278
	36	*V. stipulacea*	Wild	Sri Lanka	JP205892
	37	*V. stipulacea*	Wild	India	JP245503
	38	*V. aconitifolia*	Domesticated	India	JP245897
	39	*V. aconitifolia*^†^	Domesticated	Pakistan	JP104332
	40	*V. aconitifolia*	Wild	India	JP235416
	41	*V. aconitifolia*	Wild	India	JP245865
	42	*V. aconitifolia*	Wild	India	JP245864
	43	*V. aconitifolia*	Domesticated	India	JP245857
	44	*V. indica*	Wild	India	JP235417
	45	*V. trilobata*	Wild	Sri Lanka	JP210605
	46	*V. trilobata*	Wild	India	JP245881
	47	*V. trilobata*^†^	Wild	Sri Lanka	JP205895
	48	*V. aridicola*	Wild	Sri Lanka	JP207977
	49	*V. aridicola*	Wild	Sri Lanka	JP205896
	50	*V. aridicola*	Wild	Sri Lanka	JP205894
*Plectrotropis/Plectrotropis*	51	*V. vexillata* var. *vexillata* ^†^	Wild	Congo	JP235912
	52	*V. vexillata* var. *angustifolia*	Wild	Colombia	JP235869
	53	*V. vexillata*	Wild	Papua New Guinea	JP230747
	54	*V. vexillata*	Wild	Surinam	JP202334
	55	*V. vexillata* var. *ovata*	Wild	South Africa	JP235908
	56	*V. vexillata* var. *macrosperma*	Domesticated	Sudan	JP235905
	57	*V. vexillata*	Wild	Brazil	JP202337
	58	*V. vexillata*	Domesticated	Indonesia	JP235863
	59	*V. vexillata* var. *lobatifolia*	Wild	Namibia	JP235903
*Vigna/Catiang*	60	*V. unguiculata* subsp. *sesquipedalis*	Domesticated	Sri Lanka	JP81610
	61	*V. unguiculata*	Domesticated	Sudan	JP86879
	62	*V. unguiculata*^†^	Domesticated	Nigeria	JP86801
	63	*V. unguiculata*	Domesticated	Sudan	JP86877
	64	*V. unguiculata* subsp. *dekindtiana*	Wild	Nigeria	JP89083
*Vigna/Vigna*	65	*V. luteola*	Wild	Brazil	JP235855
	66	*V. marina* subsp. *oblonga*	Wild	Benin	JP233389
	67	*V. luteola*	Wild	Australia	JP236246
	68	*V. marina*	Wild	Japan	JP235813
	69	*V. subterranea*	Domesticated	Unknown	JP79992

*Accessions marked with ^†^ were used for the measurements of volumetric soil water content in Figure 2*.

All the accessions were evaluated in two experiments simulating a non-terminal and a terminal drought condition during the vegetative stage. In the pipe experiment for studying the effect of non-terminal drought conditions, the accessions were grown in polyvinyl chloride (PVC) pipes, wherein the soil water content decreases gradually, and the upper layer dries faster than the bottom layer. We used two different heights of pipes, producing different drought intensities at the top of the pipes. The pipe bottoms were continuously submerged in water in order to maintain soil water content in deep soil layers by capillary water uptake, so that the drought intensities did not reach the terminal level. In addition, to understand tolerance under terminal drought conditions, the plants were evaluated in a pot experiment, wherein the soil water content decreases rapidly, finally leading to dehydration.

### Pipe experiment for non-terminal drought

**Figure 1:**
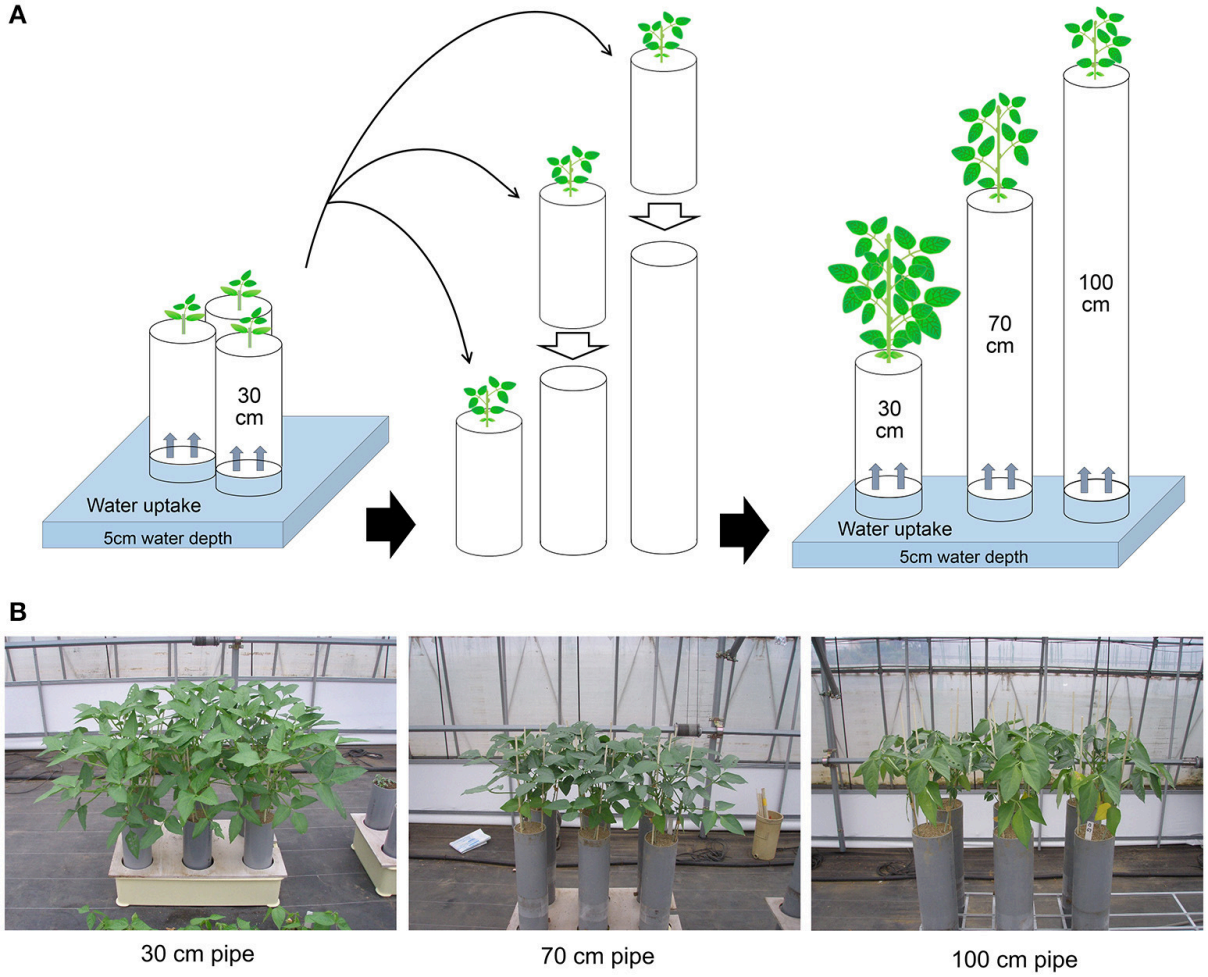
Methods for drought treatments in the pipe experiment. **(A)** All seedlings were grown in 30-cm long PVC pipes containing soil, until the first leaves had fully expanded. The drought treatments were then started by placing the 30-cm pipes onto other pipes containing the same soil and continued for 3 weeks during vegetative stage. The final heights of the pipes were either 70 or 100 cm. The control plants were grown continuously in the 30-cm pipe. Throughout the experiment, water was supplied only from the bottoms of the pipes. **(B)** Plant images after 3 weeks of drought treatment for each of the pipe heights.

The methods for drought treatments in the pipe experiment are shown in Figure 1. Pipe experiments were conducted three times, during different seasons. The sowing date for each experiment was 27 July 2013, 5 September 2013, and 22 April 2014 for the first, second, and third trial, respectively. All plants were grown in polyvinyl chloride (PVC) pipes in a greenhouse in Tsukuba, Japan. Climate conditions inside the greenhouse during the trials are summarized in Table S1. Mean values of solar radiation, daytime temperature, and daytime vapor pressure deficit were 13.5–18.5 MJ day^−1^, 28.3–32.4°C, and 12.5–17.7 hPa, respectively. Three seeds for each accession were sown in a pipe of 30-cm height and 12.5-cm diameter filled with granular culture soil (Nippi-Engei-Baido, Nihon Hiryo Co. Ltd., Tokyo, Japan) of high water permeability. The nutrient composition of the culture soil was N:P_2_O_5_:K = 0.24:3:0.24 g kg^−1^. All the pipes were placed in trays with a water depth of 5-cm. Three pipes were prepared for each accession: one for the control (30-cm), and the other two for 70- and 100-cm treatments.

When the first leaves had fully expanded (about 3 weeks after sowing), the 30-cm pipes for drought treatment were set on top of 40 or 70-cm pipes filled with the same soil, which were used as different drought intensity treatments (Figure 1A). All the pipes below were placed into water of 5-cm depth for a day before the treatment to ensure soil water conductivity between the pipes below and above. Cloth filter on the bottom of 30-cm pipe was removed and immediately put on the pipe below, then the pipes were tightly jointed using waterproof tape.

**Figure 2:**
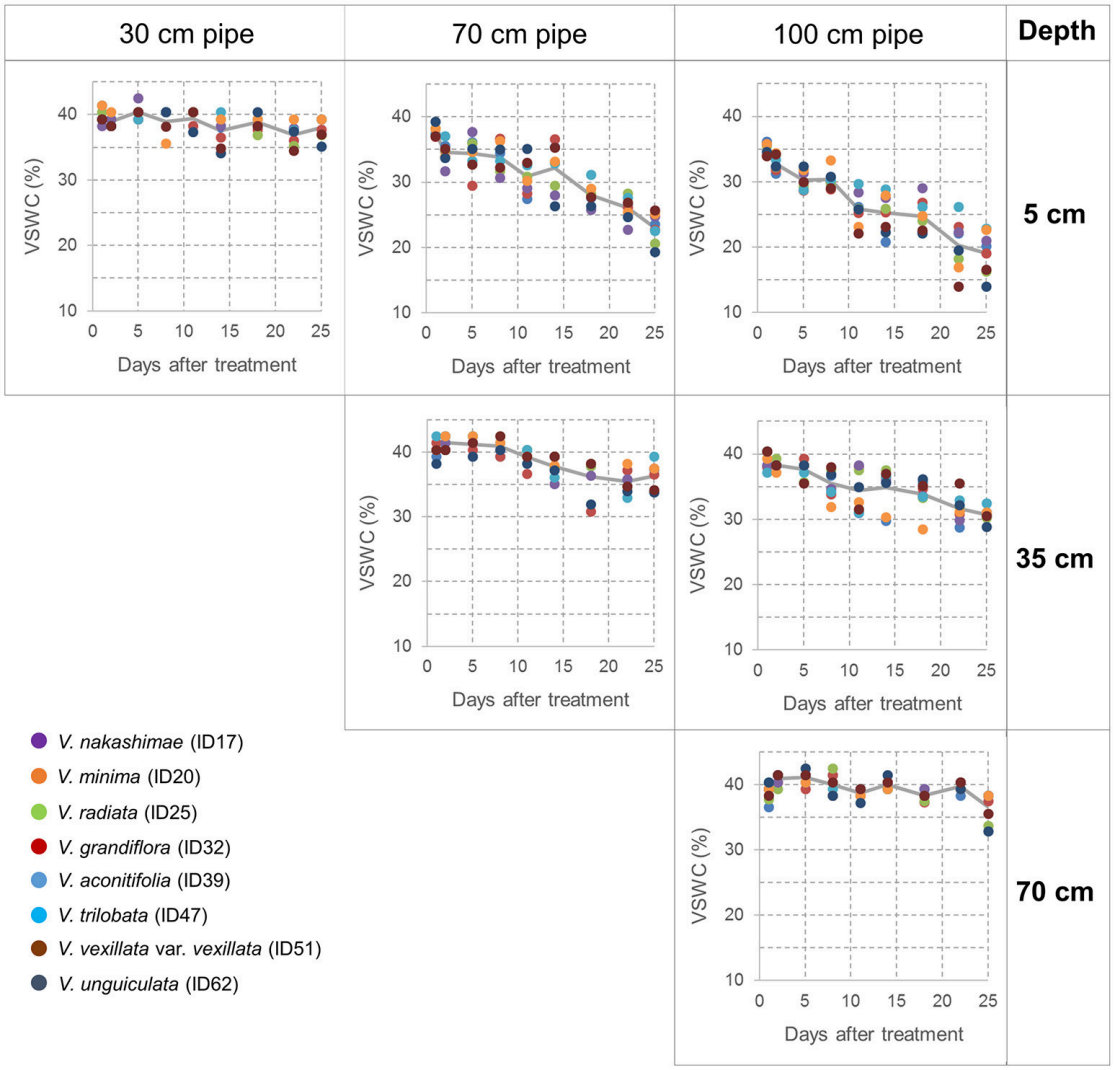
Time-course changes in the volumetric soil water content (VSWC) measured at midday during the drought treatment. Values of VSWC at 5 cm depth of 30-cm pipes (left column), at 5 and 35 cm depths of 70-cm pipes (center column), at 5, 35, and 70 cm depths of 100-cm pipes (right column). Each data point is the value for one pipe (planted with one accession), and data of eight pipes are shown at each time point. The lines represent the averages of the eight pipes.

During the drought treatment, water was continuously supplied from the bottom in the same way as before the treatment. Drought treatments were continued for 3 weeks to make drought stress limited to vegetative stage. At the end of the drought treatment, total shoots were sampled from all accessions and treatments. For the accessions of 70- and 100-cm pipe treatments, root and soil together were taken out from the pipe, and soil was carefully removed from root in water. Maximum root length was recorded as a value of three plants planted in a pipe. For each accession and treatment, the sampled shoots were dried at 80°C for 70 h and then weighed to obtain the shoot dry weight; likewise, the roots of the three plants in a pipe were combined, dried, and weighed. Data obtained from the three experimental replications, each includes three plants, were averaged and used for tolerance evaluation. Volumetric soil water content (VSWC) at different soil depths was monitored during the second trial. Time-course changes in VSWC were evaluated for a subset of eight accessions (shown in Figure 2), which had a range of shoot biomass. The VSWC was calculated from the values of gravimetric soil water content measured using a soil moisture sensor (DM-18, Takemura Denki Seisakusho, Tokyo, Japan).

### Stomatal conductance and photosynthetic rate

Stomatal conductance and photosynthetic rate were measured for the 11 accessions indicated in **Figure 9**. The measurements were done at midday (1,100–1,400 h) on 3 different days with fine weather, during the last 2 weeks of the drought treatment. For the plants of each accession in 30-, 70-, and 100-cm pipes, the stomatal conductance and photosynthetic rate of the topmost, fully expanded leaves were measured with a portable photosynthesis system (LI-6400XT, LI-COR Inc. Lincoln, NE, USA). The measurements were performed at the ambient temperature, with the photosynthetic photon flux density (PPFD) set to 1,200 μmol photon m^−2^ s^−1^, while the CO_2_ concentration was maintained at 400 ppm using a built-in CO_2_ controller. The measurements were performed after ~3 min of light exposure, when the CO_2_ gas exchange rate reached a steady state. These measurements were performed for all the three plants per pipe in each of the three experimental replicates, and the data for each accession were averaged.

### Pot experiment for terminal drought

In the pot experiment, a small plastic pot of 10-cm height and 6.5-cm diameter was used. Three seeds of each accession were sown on the same soil as the pipe experiments. All the pots were soaked in water of 2-cm depth, up to the time of drought onset. About 3 weeks after planting, when the first leaf was fully expanded in all accessions, the water application was terminated and the pots were transferred onto a mesh table to accelerate soil drying. The plants were kept for a month without any water application. A longer treatment was needed than pipe experiment because some accessions had survived and maintained the greenness even at 3 weeks after treatment. The experiment was conducted three times, in parallel with the pipe experiments. Climate conditions during the experiment was same as the pipe experiment (Table S1). Data obtained from the three experimental replications, each includes three plants per pot, were averaged and used for tolerance evaluation. Changes in VSWC at a soil depth of 5-cm during the drought treatment in the second trial are shown in Figure S1.

Decrease in green leaf area was measured over time for tolerance evaluation according to generally used dry-down-method (Iuchi et al., 2000). To quantify the extent of plant damage, whole-plant images were taken every 3 days, from 1 m above the plants, using a digital camera (μ 730, Olympus, Tokyo, Japan). The pictures were taken alongside a scale, and the green leaf area was estimated using an image analysis software (ImageJ version 1.46, National Institutes of Health; http://rsb.info.nih.gov/ij/). During data analysis, the relative reduction in green leaf area was calculated, with the value at drought onset as 1 (initial value). The number of days until 80% decrease in the green leaf area was used as Days-to-wilt (DTW), the indicator of tolerance. Image analysis was done separately for the three experimental replicates, and the values of DTW were averaged.

### Leaf temperature

Leaf surface temperature was measured using infrared thermography (G100EX, Nippon Avionics Co. Ltd., Tokyo, Japan). Thermal images were taken on 3 different days with fine weather, during the last 2 weeks of drought treatment in trial 2. By using analysis software (InfRec Thermography Studio 5.1, Nippon Avionics Co. Ltd.), the surface temperatures of the topmost fully expanded leaves were obtained and the data for each accession were averaged. Temperature difference between ambient air and leaf surface (T_a_-T_s_) was calculated to estimate leaf transpiration representing plant water status.

### Statistical analysis

To test significant differences of VSWC among the pipes, levels of soil depth or accessions in the pipe experiment, analysis of variance (ANOVA) was applied. This analysis was performed for each of the measurement dates during the drought treatment using statistical software R version 3.3.1.

In the pipe experiment, each of the values for shoot and root dry weight in the 70- and 100-cm pipes was divided by the corresponding control value (dry weight in 30-cm pipe) to generate relative shoot dry weight (RSW) and relative root dry weight (RRW). We used RSW as a tolerance indicator to evaluate vegetative growth retardation. Data obtained from the three experimental replications, each includes three plants, were averaged and applied for the following cluster analysis.

Manhattan distances computed from the values of RSW on 70- and 100-cm pipes were then subjected to hierarchical clustering, to group together the accessions with similar shoot growth response against drought treatments. Cluster analysis was performed using the complete linkage method. Then, to detect the significant differences due to drought effects on shoot and root dry weight among the clustered groups or drought treatments, ANOVA was performed. Among the groups, comparisons were carried out by Tukey's HSD test. The cluster analysis and Tukey's HSD test were performed using the same statistical software as the ANOVA.

## Results

### Soil water conditions in the pipe and pot experiment

Time-course changes in VSWC for the second trial of the pipe experiment are shown in Figure 2. The longer pipes had deeper water tables and lower soil water content in the upper soil layer. VSWC data during the first and third trials were not collected, but the climate conditions were not largely different among the seasons (Table S1) and the experimental conditions could be considered as similar. VSWC at the top (5-cm depth) of the 70- and 100-cm pipes decreased gradually and reached the values of 23 and 19%, respectively, at the end of the experiment. Soil drying rate in the upper layer clearly differed, depending on the height from the bottom. VSWC at the pipe top decreased faster in the 100-cm pipes than in the 70-cm pipes. On the other hand, at about 30-cm from the bottom (water table), which corresponded to a depth of 5-, 35-, and 70-cm in the 30-, 70-, and 100-cm pipes, respectively, the average values of VSWC were >35%, throughout the experimental period. At 35 cm depth in the 100-cm pipes, VSWC also decreased gradually, but the final value was higher than 30%. Figure 2 shows the differences in VSWC among the accessions of different shoot biomass but the differences were not statistically significant at all the measurement dates (Table 2), while significant differences were detected for different pipe heights and levels of soil depth throughout the experimental period.

**Table 2:** Summary of analysis of variance for VSWC measured at different 9 days during drought treatment in the pipe experiment.

	**Days after treatment**								
	**1**	**2**	**5**	**8**	**11**	**14**	**18**	**22**	**25**
Pipe height	0.02^*^	0.261^ns^	0.01^*^	0.01^*^	<0.01^**^	<0.01^**^	<0.01^**^	<0.01^**^	<0.01^**^
Soil depth	<0.01^**^	<0.01^**^	<0.01^**^	<0.01^**^	<0.01^**^	<0.01^**^	<0.01^**^	<0.01^**^	<0.01^**^
Accession	0.88^ns^	0.97^ns^	0.69^ns^	0.95^ns^	0.77^ns^	0.29^ns^	0.81^ns^	0.35^ns^	0.33^ns^
Pipe height × Accession	0.99^ns^	1.00^ns^	0.99^ns^	0.81^ns^	0.83^ns^	0.10^ns^	0.57^ns^	0.16^ns^	1.00^ns^
Soil depth × Accession	0.71^ns^	1.00^ns^	0.99^ns^	0.79^ns^	0.75^ns^	0.35^ns^	0.49^ns^	<0.01^**^	1.00^ns^

The significant effects of pipe height, soil depth and accession on VSWC were shown in p-values. The effects of interactions between pipe height × accession and soil depth × accession were also shown. In the ANOVA table, **, *, and ns represent p < 0.01, p < 0.05, and non-significant, respectively.

Compared with the pipe experiment, extreme and raid soil drying was observed in the pot experiment. The average values of VSWC decreased to 25% within the first 5 days after treatment and 15% at 10 days after treatment. The VSWC differed among the accessions but the differences were not statistically significant.

### Temperature difference between ambient air and leaf surface

**Figure 3:**
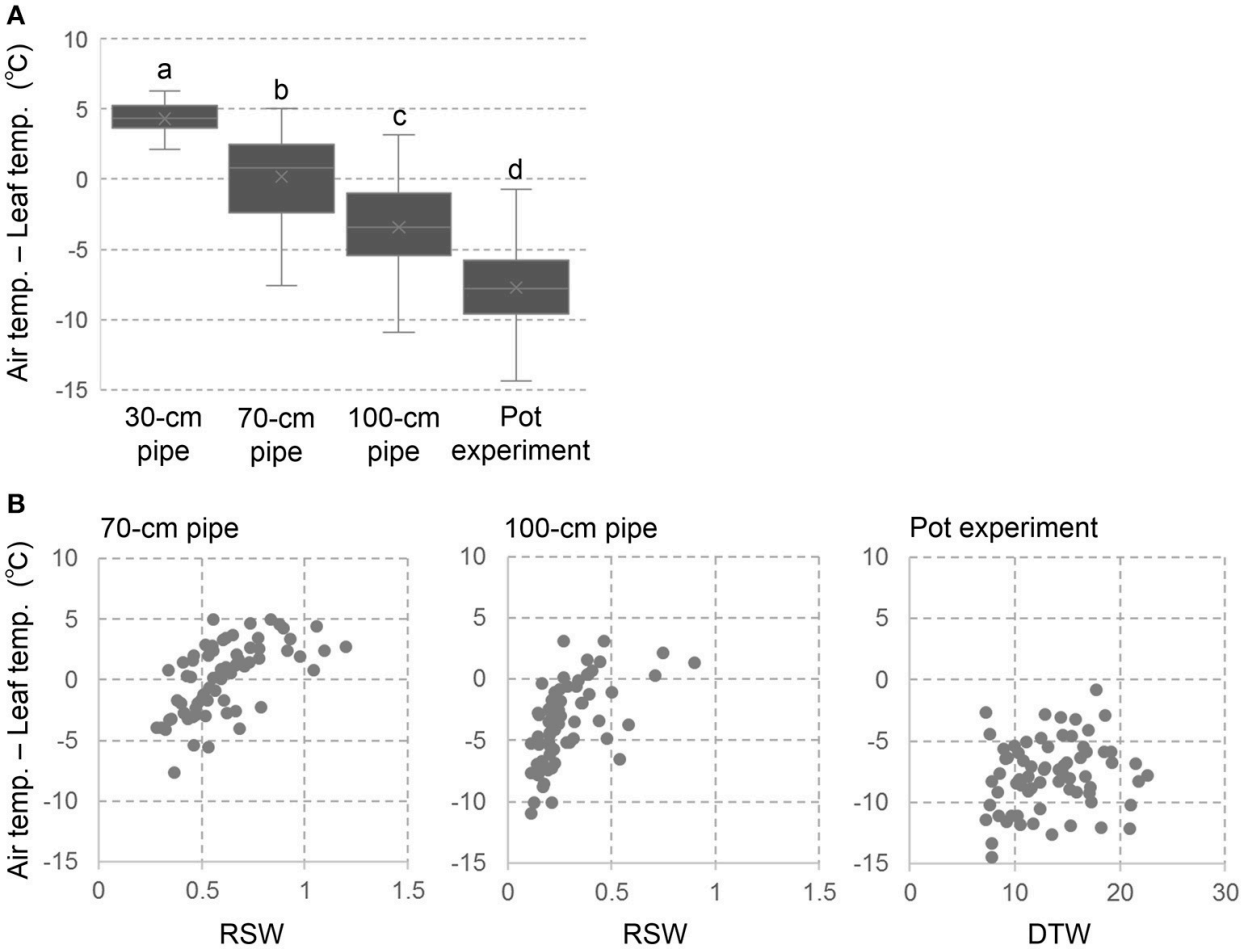
Temperature difference between ambient air and leaf surface (T_a_-T_s_) in the pipe and pot experiments. **(A)** box plots of T_a_-T_s_ for the 69 accessions. Different letters represent significant differences at *p* < 0.05 level. **(B)** Relationships between T_a_-T_s_ and RSW in the pipe experiment or DTW in the pot experiment.

The temperature difference between ambient air and plant canopy has been used as an index of plant water status (Takai et al., 2010; Zia et al., 2013). We applied this method to single plant level as using leaf temperature instead of canopy temperature. The negative value of T_a_-T_s_ indicates decreased leaf transpiration and poor water status due to drought stress, while the positive value indicates good plant water status with high leaf transpiration. The drought effects on T_a_-T_s_ was clearly shown in Figure 3A where negative values of T_a_-T_s_ were observed in the severer drought conditions. In the 30-cm pipe, the values in all the accessions were positive and the genotypic variations were minimum level. On the other hand, approximately half of the accessions in the 70-cm pipes and most accessions in the 100-cm pipes were negative values. Severe negative values were observed in the pot experiment indicating extremely poor plant water status. In all the drought conditions of pipe and pot experiments, large genotypic variations in T_a_-T_s_ were observed.

Figure 3B shows the relationships between T_a_-T_s_ and RSW in the pipe experiment or DTW in the pot experiment. In both of the 70- and 100-cm pipes, accessions of high RSW showed positive values in T_a_-T_s_. However, values in T_a_-T_s_ were largely varied in the accessions of lower RSW, and no relationship was observed between T_a_-T_s_ and RSW among them. Also in the pot experiment, T_a_-T_s_ was not related with DTW.

### Shoot and root dry weights in the pipe experiment

**Figure 4:**
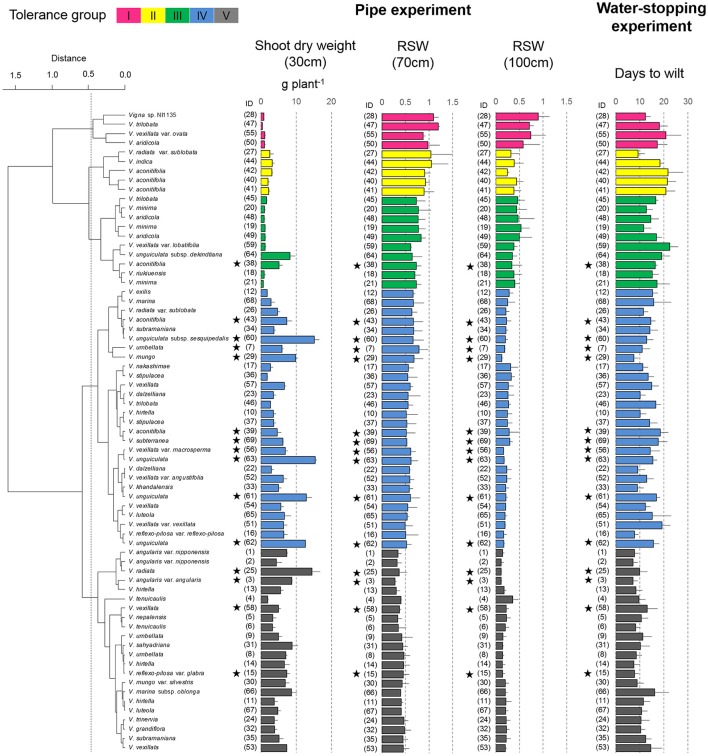
Drought response of shoot dry weights in the pipe experiment and days-to-wilt (DTW) in the pot experiment. The shoot dry weight for the 30-cm pipe and the relative ratios (RSW) for 70 and 100-cm pipes are shown in the cluster tree. Data for shoot dry weight, RSW, and DTW are shown as averages ± standard errors for the three trials. The five groups identified by the cluster analysis are indicated using different colors. Group I, magenta; Group II, yellow; Group III, green; and Group IV, blue; Group V, black. Stars indicate domesticated accessions.

**Figure 5:**
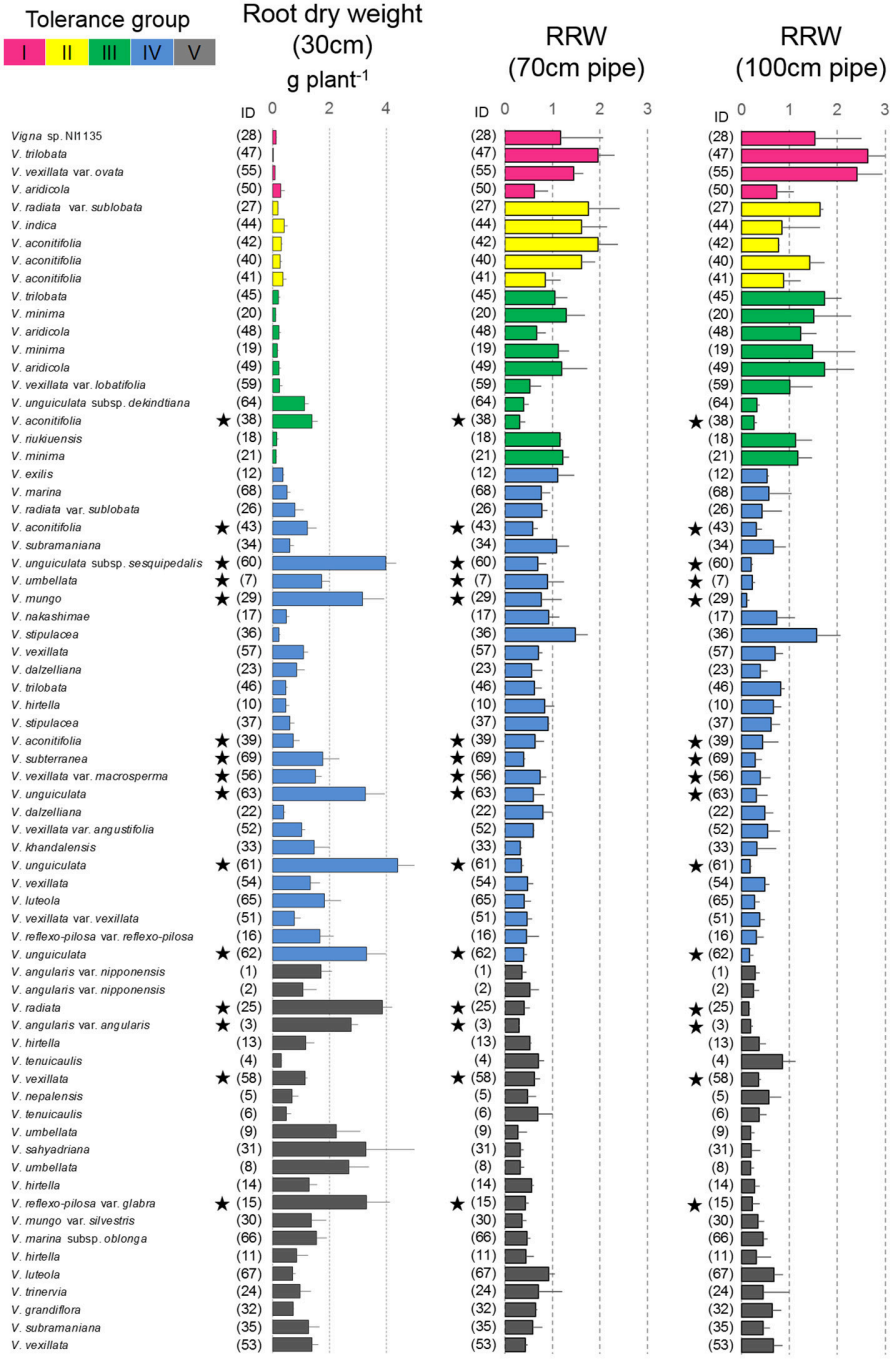
Drought response of root dry weights in the pipe experiment. The root dry weight for 30-cm pipes and the relative ratios (RRW) for 70 and 100-cm pipes are shown. Data of RRW are the averages ± standard errors for the three trials. The five groups identified by cluster analysis are indicated using different colors, same as shown in Figure 4. Stars indicate domesticated accessions.

In 30-cm pipes, the shoot and root dry weights varied among the 69 accessions, ranging from 0.4 to 15.4 g per plant (Figure 4) and from 0.03 to 4.4 g per plant (Figure 5), respectively. Figure 3 also shows the RSW for the 70- and 100-cm pipes. The average RSW for the 69 accessions were 0.60 and 0.28 in the 70- and 100-cm pipes, respectively. Compared with the shoot, the RRW was less affected by the drought treatments; the mean RRW was 0.76 and 0.67 in the 70- and 100-cm pipes, respectively (Figure 5). A high correlation in the relative dry weights was observed between the shoot and root (*r* = 0.74 and 0.84 at *p* < 0.01 level for 70- and 100-cm pipes, respectively), and between the 70- and 100-cm pipes (*r* = 0.75 and 0.78 at *p* < 0.01 level for shoot and root, respectively).

**Figure 6:**
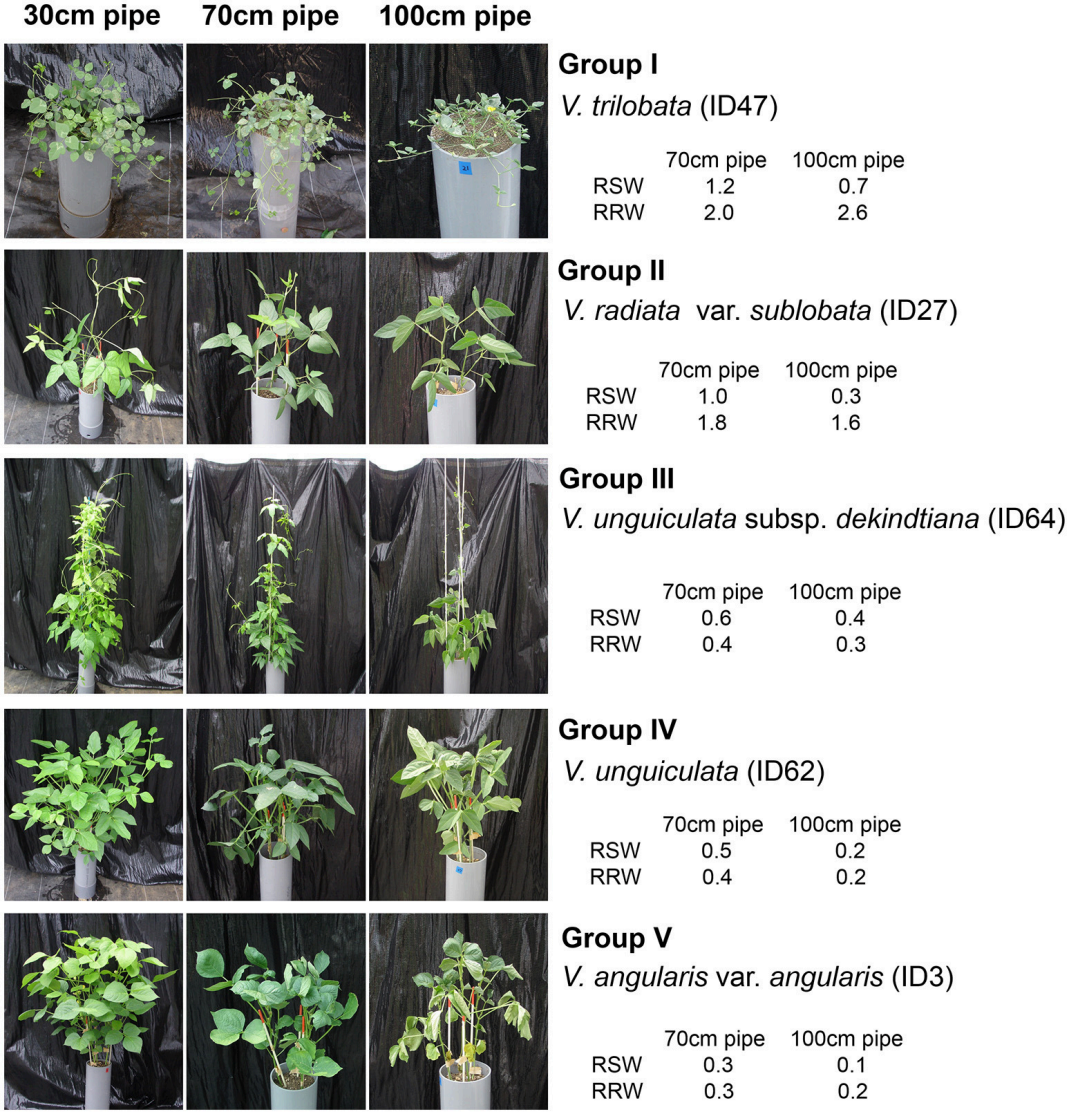
Photos of the accessions representing the five drought-tolerant groups. Photos were taken at the same time as plant sampling, 3 weeks after the drought treatment.

**Figure 7:**
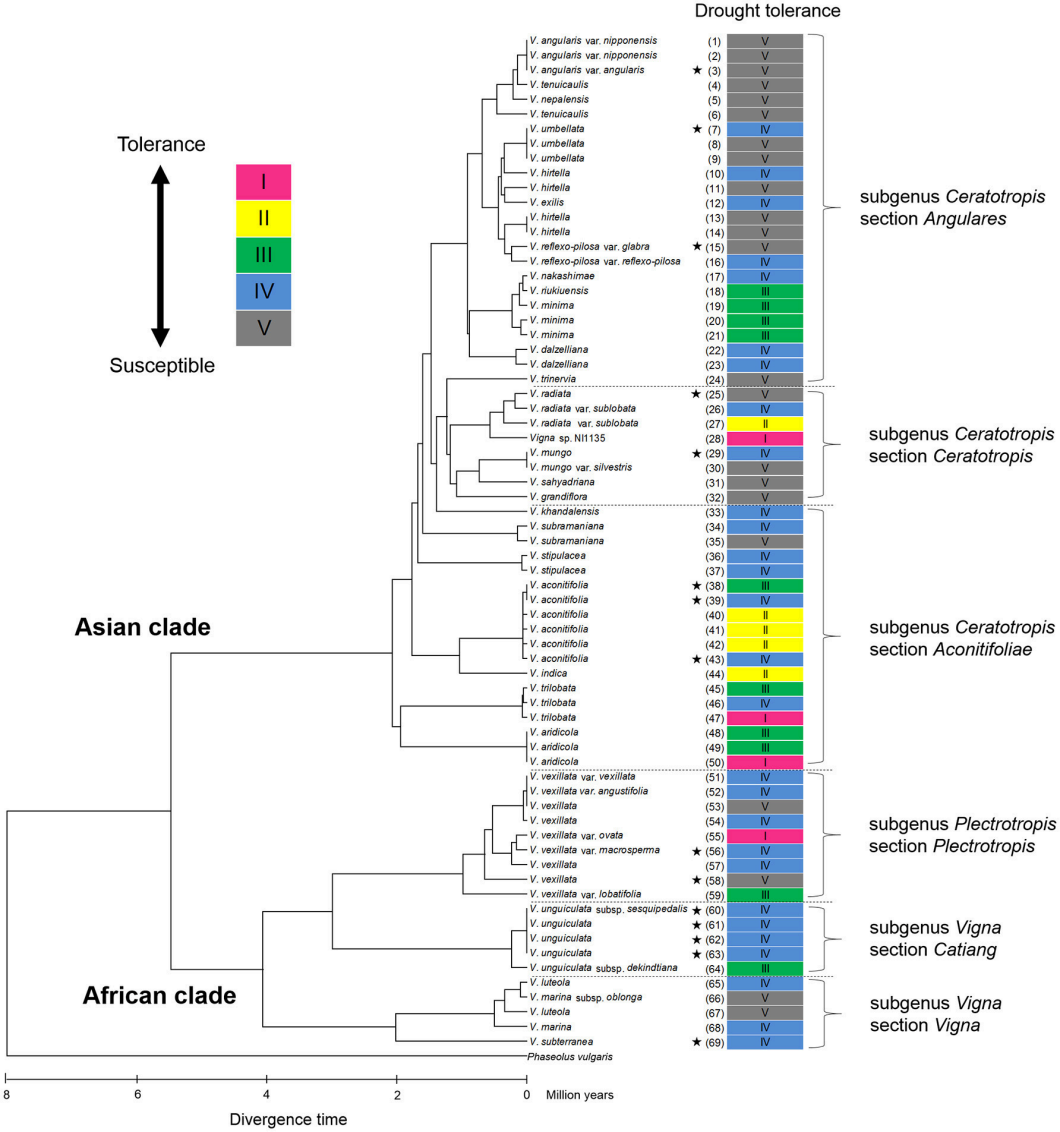
Distribution of drought tolerance in phylogenic tree of the 69 accessions in genus *Vigna*. The phylogenetic tree was obtained from our former study (Iseki et al., 2016). The original figure of the phylogenic tree is free for use according to “Creative Commons Attribution (CC BY) license” (https://creativecommons.org/licenses/by/4.0/). The under scale represents divergence time in million years. Stars indicated domesticated accessions.

Cluster analysis classified the 69 accessions into five groups, Group I (tolerant), Groups II and III (medium tolerant), Group IV (medium susceptible), and Group V (susceptible), based on the RSW in 70- and 100-cm pipes (Figure 6). In Figure 7, the position of each tolerance group accession is shown on a phylogenetic tree used in one of our former studies (Iseki et al., 2016). Based on this, we show that the tolerant accessions of group I–III were widely distributed among the subgenuses of *Vigna*.

Group I accessions showed the highest average value of RSW in the 70- and 100-cm pipes (1.04 and 0.73 in the 70- and the 100-cm pipes, respectively; Table 3). Group II accessions showed a high average RSW in the 70-cm pipe (0.97), but it largely decreased in the 100-cm pipe (0.35). Group III accessions showed a medium decrease in the average RSW for the 70-cm pipe (0.73), but the value for the 100-cm pipe (0.43) was higher than that of Group II. Group IV accessions also showed a medium decrease in the RSW for the 70-cm pipes and the average value was less than that of Group III. Average RSW in the 100-cm pipe of Group IV decreased to 0.23. Group V accessions were the most susceptible to drought, with average RSW of 0.40 and 0.18 in the 70- and the 100-cm pipes, respectively.

**Table 3:** Response of the shoot and root dry weights in the pipe experiment.

**Group**	**Shoot**			**Group**	**Root**		
	**Dry weight (g plant^−1^)**	**Relative ratio to control**			**Dry weight (g plant^−1^)**	**Relative ratio to control**	
	**30 cm**	**70 cm**	**100 cm**		**30 cm**	**70 cm**	**100 cm**
I	0.8b	1.04a	0.73a	I	0.13ab	1.30ab	1.83a
II	2.6ab	0.97a	0.35c	II	0.31ab	1.56a	1.11b
III	2.2b	0.73b	0.43b	III	0.40b	0.89bc	1.16b
IV	6.2a	0.59c	0.23d	IV	1.43a	0.69cd	0.47c
V	6.0a	0.40d	0.18d	V	1.58a	0.50d	0.39c
Group		^**^		Group		^**^	
Water condition		^**^		Water condition		ns	
Interaction		^**^		Interaction		^**^	

Values are the averages for each group. For each column, different letters represent significant differences at p < 0.05 level. In the ANOVA table, ** and ns represent significance at p < 0.05 level and not significant, respectively.

Most of the accessions in Groups I–III were wild accessions, with smaller shoot and root dry weights compared with those of Groups IV and V (Table 3). Many accessions in Groups I–III showed an increased root dry weight in both 70- and 100-cm pipes. Especially, in the 100-cm pipes, RRW for *V. trilobata* (ID47) and *V. vexillata* var. *ovata* (ID55) showed the highest values (2.4 and 2.6, respectively; Figure 5). RRW of most accessions in Groups IV and V was <1.0 in both 70- and 100-cm pipes. Even the domesticated accessions known for drought tolerance, such as cowpea (ID60, 61, 62, 63), moth bean (ID39, 43), and bambara groundnuts (ID69), were included in Group IV.

### Root length

**Figure 8:**
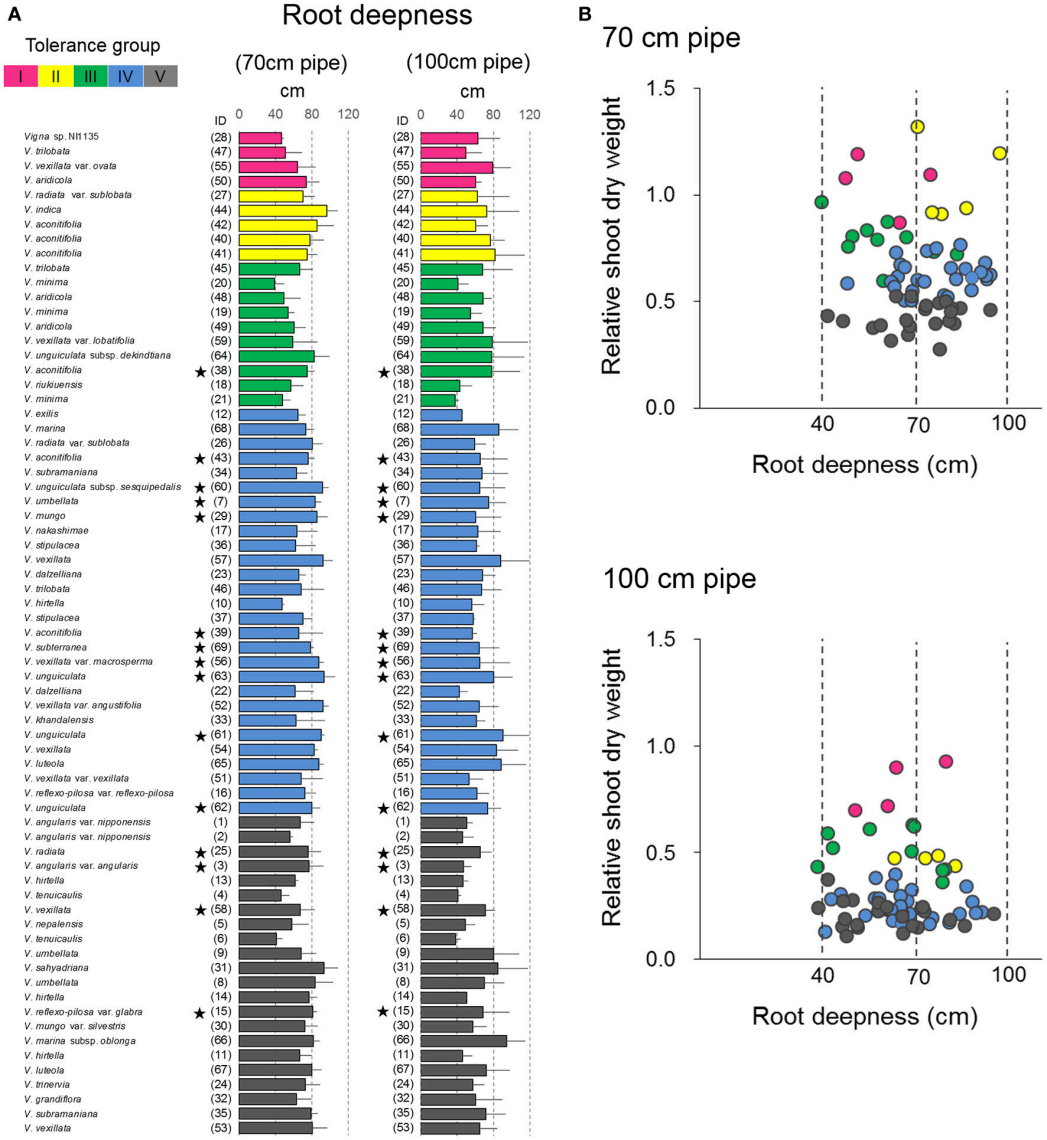
Drought response of root depth in the 69 accessions. **(A)** The maximum root length in the 69 accessions and **(B)** relationships between the maximum root length and RSW in the pipe experiment. Data of root length are the averages and the error bars are standard errors for the three trials. The five groups identified by cluster analysis are indicated in different colors as shown in Figure 4. Stars indicate domesticated accessions.

In the pipe experiment, a large variation was observed in the maximum root length (Figure 8A), which is generally considered one of the most important traits for drought tolerance (Cattivelli et al., 2008). However, our results showed almost no correlation between RSW and root length in both 70- and 100-cm pipes (Figure 8B). For the 70-cm pipes, all accessions of Groups IV and V had a root length >40 cm, which made them deep enough to reach the soil of high VSWC (Figure 2). For the 100-cm pipes, the roots of many accessions in Groups IV and V reached the soil of high VSWC at the depth of 70 cm, although their RSW values were severely low. On the other hand, some accessions in Groups I–III had short roots but retained high RSW (Figures 4, 8A).

### Stomatal conductance and photosynthetic rate

**Figure 9:**
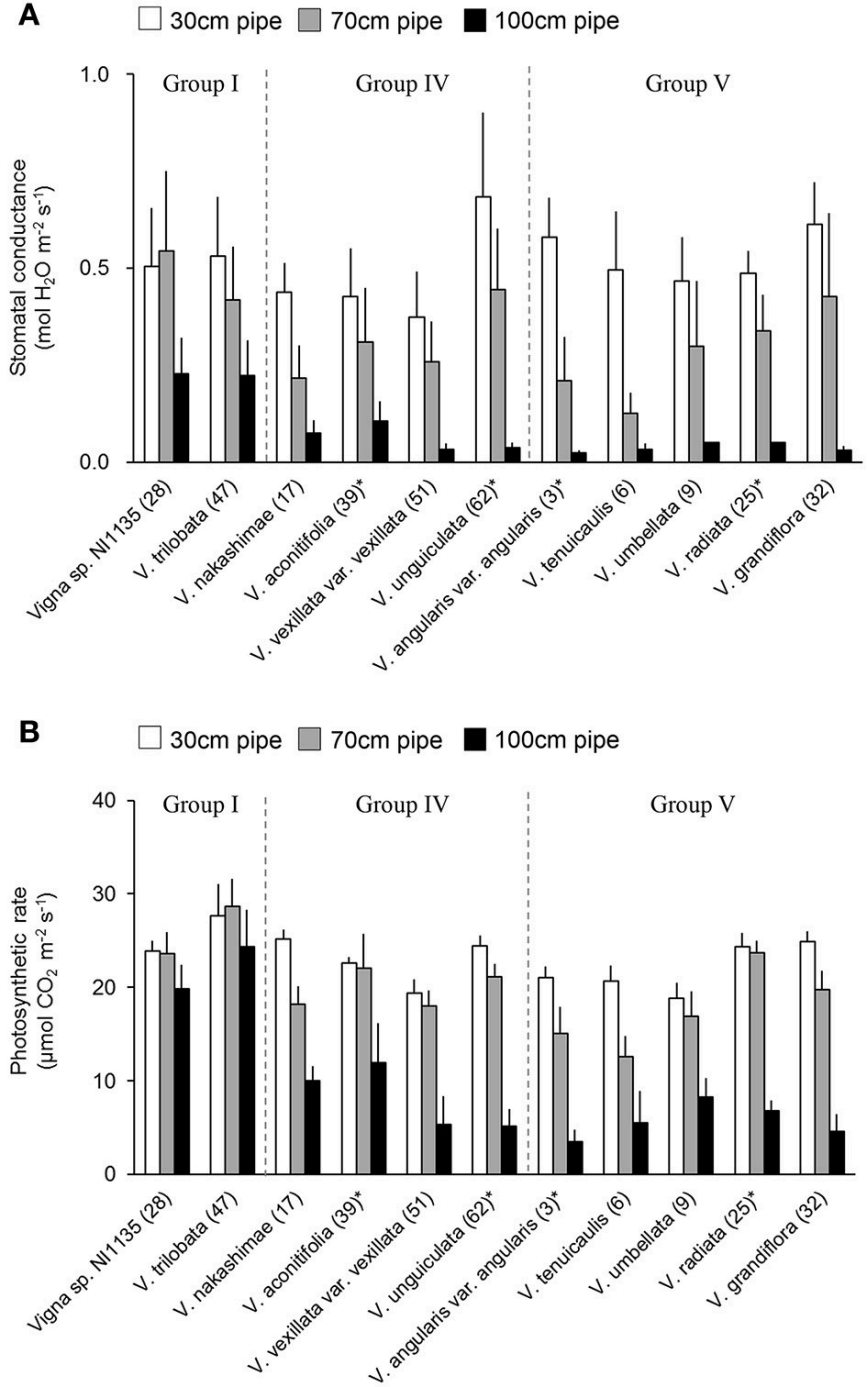
Stomatal conductance **(A)** and the photosynthetic rate **(B)** of selected 11 accessions in tolerant (Group I) and susceptible (Group IV and V) groups. Bars are average ± standard errors for the three trials. Asterisks indicate domesticated accessions. White, gray and black bars represent 30-cm, 70-cm and 100-cm pipes, respectively.

Stomatal conductance was measured for accessions in tolerant (Group I) and susceptible (Group IV and V) groups. Because the stomatal closure is known as early response to drought stress (Medrano, 2002), it can be used as an indicator of drought sensitivity. The leaf stomatal conductance are shown in Figure 9A. In the 70-cm pipes, higher stomatal conductance (>0.4 mol H_2_O m^−2^ s^−1^) was observed not only in Group I accessions but also in *V. unguiculata* (ID62, Group IV) and *V. grandiflora* (ID32, Group V). In contrast, a lower stomatal conductance was observed in *V. nakashimae* (ID17) in Group IV, and *V. angularis* var. *angularis* (ID3) and *V. tenuicaulis* (ID6) in Group V. In the 100-cm pipes, while *Vigna* sp. NI1135 (ID28) and *V. trilobata* (ID47) maintained the stomatal conductance around 0.25 mol H_2_O m^−2^ s^−1^, other accessions had decreased values to <0.1 mol H_2_O m^−2^ s^−1^.

Photosynthetic rate was measured at the same time with stomatal conductance measurement. Photosynthetic rate was less sensitive to drought in comparison with stomatal conductance but it is closely related with biomass production. In contrast with stomatal conductance, the photosynthetic rate was less affected in the 70-cm pipes, with the values showing no great change in most of the 11 accessions (Figure 9B). Small decreases were observed in *V. nakashimae* (ID17) in Group IV, and *V. angularis* var. *angularis* (ID3), *V. tenuicaulis* (ID6), and *V. grandiflora* (ID32) in Group V. In the 100-cm pipes, the photosynthetic rate of *Vigna* sp. NI1135 (ID28) and *V. trilobata* (ID47) in Group I remained around 20 μmol CO_2_ m^−2^ s^−1^, whereas it decreased to 10 μmol CO_2_ m^−2^ s^−1^ in all other accessions.

### Days-to-wilt (DTW) in the pot experiment

The plant survivability was evaluated using DTW in the pot experiment. Values of DTW in the 69 accessions varied from 7.2 to 22.5, as shown in Figure 4. DTW > 20 were observed only for *V. vexillata* var. *ovata* (ID55) in Group I, *V. aconitifolia* (ID40–42) in Group II, and *V. vexillata* var. *lobatifolia* (ID59) in Group III. Accessions with a higher DTW than the average (13.4) were widely observed throughout Groups I–V. Accessions with DTW < 10 days were mainly observed in Group V. It should be noted that even in the tolerant groups (I–III), *Vigna* sp. NI1135 (ID28), *V. radiata* var. *sublobata* (ID27), and *V. minima* (ID19, 22) showed a short DTW (<13).

## Discussion

### Drought-tolerant accessions in the pipe experiment of non-terminal drought

For the evaluation in the pipe experiment of non-terminal drought, we used RSW as a tolerance index to evaluate ability to maintain shoot growth. Among the 69 accessions, four accessions of Group I were classified as the most tolerant accessions (Figure 4). Group I accessions could maintain RSW > 0.5 even in the 100-cm pipes. Under this condition, only *Vigna* sp. NI1135 (ID28) showed an RSW > 0.8 (Figure 4). The Group I accessions also showed higher stomatal conductance and photosynthetic rate (Figure 9), suggesting that they had high adaptability to maintain water uptake and leaf functions related to photosynthesis.

Among the Group I accessions, *Vigna* sp. NI1135 (ID28) was originated from the Himalayan highland of North India. *V. trilobata* (ID47) and *V. aridicola* (ID50) were originated from dry sandy beach and inland dry sandy grassland of Sri Lanka (Tomooka et al., 2002). *V. vexillata* var. *ovata* (ID55) was sourced from the southeastern coastal area of South Africa. The ability to maintain a higher RSW might be related to their origin of dry, sandy habitats. Especially for ID28 and ID47, RSW was >1.0 in the 70-cm pipes indicating this soil water condition was more favorable than that in the 30-cm pipes. Because VSWC in 30-cm pipes was continuously >35% throughout the experiment, soil moisture might be in excess for the accessions from dryland. In addition, we identified relatively tolerant accessions in Groups II and III, which showed also higher RSW, especially in the 70-cm pipes. These accessions could serve as promising sources for improving vegetative growth under non-terminal drought conditions, considering the wide cross-compatibility reported in *Vigna* species (Tomooka et al., 2011). Further, these accessions could also serve as effective genetic source to clone genes involved in drought tolerance.

### Drought tolerance of *vigna* crops in the pipe experiment

All the domesticated accessions (nine species) were classified in either Group IV or V, medium susceptible or susceptible groups, except *V. aconitifolia* (moth bean, ID38) in Group III. Although cowpea (*V. unguiculata*, ID61–63), bambara groundnuts (*V. subterranea*, ID69), and moth bean (*V. aconitifolia*, ID39, 43) are the crops widely cultivated in drought-prone regions, our results indicate that they had rather lower RSW. On the other hand, the tolerant wild accessions were widely distributed for subgenuses of *Vigna*, and phylogenic tree of drought tolerance indicated that the tolerance of domesticated species would be improved through cross-breeding with genetically close wild accessions (Figure 7).

Cowpea is one of the most important crops cultivated mainly in tropical and sub-tropical region of sub-Saharan Africa (Agbicodo et al., 2009). As drought stress is a major production constraint in these regions, further improvement in drought-tolerant traits is needed. In this study, we used some cowpea accessions (ID61–63) reported as drought-tolerant in former studies (Iuchi et al., 2000; Agbicodo et al., 2009). However, these accessions were found less in RSW compared to an accession, *V. unguiculata* subsp. *dekindtiana* (ID64), a wild relative of cowpea. Likewise, the vegetative stage drought tolerance of domesticated *V. aconitifolia*, cultivated mainly in South Asia, could also be improved using their wild conspecific ancestors (IDs 40–42 in Group II).

The important Asian legume crops, mungbean (*V. radiata* ID25) and azuki bean (*V. angularis* ID3) were classified into Group V, the most susceptible group. To improve the vegetative stage drought tolerance in mungbean, *Vigna* sp. NI1135 (ID28 in Group I) and *V. radiata* var*. sublobata* (ID27 in Group II) might be useful because these accessions are cross-compatible with mungbean. As for azuki bean, the wild ancestral accessions (*V. angularis* var. *nipponensis* ID1, 2) were highly drought-susceptible (Group V). However, there are cross-compatible wild relatives (*V. minima*, ID19–21 and *V. riukiuensis*, ID18) in Group III. These accessions could be used to improve the vegetative stage drought tolerance of the cultivated azuki bean.

### Mechanisms of maintaining RSW in the pipe experiment

In the pipe experiment, it is likely to see primary effects of shoot biomass on soil water content. Among the 69 accessions of variations in shoot biomass, plants with large shoot biomass might rapidly decrease soil water content due to high water consumption while plants with a small shoot biomass might have low water consumption and slowly decrease soil water content. However, differences in VSWC among the accessions were insignificant level (Table 2) indicating soil water conditions in the pipes were mainly determined by the height from the water table due to gravimetric effect and capillary effect.

Plant water status estimated from T_a_-T_s_ was largely differed among the accessions of different shoot biomass although there were no correlations between T_a_-T_s_ and VSWC (Figure S2). This means that differences in shoot biomass affected to plant water status rather than the soil water content in the pipe experiment, and finally the accessions with high plant water status showed high RSW (Figure 3B). This is consistent with the fact that most accessions of Groups I–III had a low shoot dry weight (<4 g per plant in the 30-cm pipes, Figure 4).

It seems that a low shoot biomass is inevitable for good plant water status and achieving higher vegetative stage drought tolerance. However, high values in T_a_-T_s_ were also observed in the accessions with low RSW (Figure 3B). In addition, high RSW was not always shown in accessions of low shoot biomass especially when the shoot dry weight was in the range of 4–16 g per plant, or among all the susceptible accessions of Groups IV and V (Figure S4). On the other hand, some accessions in Groups IV and V also had a small shoot biomass. The large decrease in stomatal conductance in a small plant (*V. tenuicaulis*, ID6) of Group V indicated that even small plants could face leaf water scarcity in the 70- and 100-cm pipes (Figure 9). Therefore, tolerance of the small accessions in Groups I–III could not be explained only by low water consumption accompanied with small shoot biomass. This notion also indicates that RSW of the domesticated accessions in Groups IV and V can be improved to some extent without any reduction in the plant body size.

We expected that deep rooting is another important trait for maintaining plant shoot growth (Price et al., 2002). However, no correlation between RSW and the maximum root length was found (Figure 8B). Especially in the 70-cm pipes, the roots of more than half the accessions reached out from the bottom of the pipes (>70 cm in maximum root length), where water was always present. Most of these accessions could not retain shoot growth and many showed RSW of <0.5. In addition, we noted that VSWC was >30% at the depth of 35 cm in the 100-cm pipes (Figure 2), and even the accession with the shortest roots had ~40-cm long roots. These results indicate that low-RSW accessions could not absorb enough water for shoot growth from even 35 cm soil depth. As such, only deep rooting could not fully explain the variation in RSW and there must be other reasons.

As one of the other reasons, we must consider root biomass and distribution, because it is expected that the accessions allocating larger amounts of root in the deep soil layer could maintain higher water absorption. In relation to this, we found an increase in the root biomass in response to soil drying (Figure 5). In contrast with Groups IV and V, wherein most accessions decreased the root dry weight in the 70- and 100-cm pipes, many accessions of Groups I–III increased the root dry weight, although their shoot dry weights either decreased or stayed at the same level as the control condition. Especially high amounts of root increase were observed in *V. trilobata* (ID47) and *V. vexillata* var. *ovata* (ID55), which had more than twice the root dry weight in the 100-cm pipes, compared with the 30-cm pipes. The increase in root biomass could be explained by ABA accumulation in the root, which simultaneously inhibits shoot growth to some extent (Blum, 1996). These accessions might change dry matter allocation in the early phase of soil drying, and the increased root biomass could supply enough water to maintain the leaf water status in the 70- and 100-cm pipes in the later soil drying.

In addition, we have noticed that some accessions in Groups I–III showed higher shoot/root ratio in the 30-cm pipes (Figure S3), indicating that the shoot biomass was established by water supply from relatively small amounts of roots. This can be explained by two mechanisms. First is water saving traits, such as low stomatal conductance and low leaf hydraulic conductance in the shoot (Sinclair et al., 2008; Lawson and Blatt, 2014). In addition, root traits, such as number and diameter of xylem vessels and root suberization, also serve to conserve water (Lynch et al., 2014). They allow high water productivity, enabling dry matter production with less water consumption. Second is water capturing by roots, such as high root length density coupled with high root hydraulic conductance (Steudle, 2000). They allow enough water uptake for their water consumption with small root quantity. The high dry matter allocation into roots during early soil drying might be assured by the high shoot/root ratio under non-stressed (30-cm pipe) condition, representing a capacity to produce shoot biomass with less amount of root.

As such, several factors are conceivable for the variation in RSW but factors responsible for tolerance might be differ from accession to accession. Further studies are needed to clarify the tolerance mechanisms in each of the accessions.

### Tolerance in the pot experiment of terminal drought

In the pot experiment of terminal drought condition, tolerance evaluation was made depending on DTW calculated from remaining leaf green area. After the cease of water application, leaf expansion stopped and the leaves started to wilt. Due to extreme and rapid soil water scarcity, differences in root characteristics might not be effective under this condition. Plant water status represented by T_a_-T_s_ was largely different among the accessions but it was not related with VSWC (Figure S2) and DTW (Figure 3B). This might be because of the severe soil dehydration where the most important trait for long DTW seemed to be water saving to reduce plant water consumption rather than water capturing from soil.

However, reduction in leaf transpiration simultaneously induces increase in leaf temperature due to a lack of latent heat flux. Because high leaf temperature damages cell membranes and affects metabolic activities and finally causes leaf senescence (Xu and Zhou, 2006), heat tolerance is also an important trait for long DTW. Therefore, we focused on leaf morphologies related with water saving and heat tolerance as traits corresponding to long DTW.

We selected five accessions with DTW > 20 days as the most tolerant accessions. All the five accessions had specific morphological leaf traits. Leaf lobing reduces the distance from the vein to the leaf edge, which decreases water loss by transpiration or during inter-cellar transfer, and serves to maintain transpiration per unit leaf area by avoiding leaf temperature increase (Nicotra et al., 2011). The deeply lobed leaves were present in the wild accessions of *V. aconitifolia* (ID40, 41, 42), which inhabit sandy arid regions. Small leaves are known to increase the heat flux from the leaf surface, because smaller-sized leaves have thinner boundary layer, causing rapid heat conversion (Nicotra et al., 2011). Small leaves were observed in *V. vexillata* var. *ovata* (ID55) living in sandy coast. The high-density hairiness of leaves is also known as an adaptive trait that reduces the heat load by restricting light absorbance (Rotondi et al., 2003). This trait is seen in *V. vexillata* var. *lobatifolia* (ID59) grown in the Namib Desert. These morphological traits might have evolved during adaptation to the arid environment. Other traits such as cuticle thickness and stomatal response are also important traits to achieve long DTW (Kosma et al., 2009; Negin and Moshelion, 2016).

Although all the above five accessions showed low shoot biomass in the pipe experiment, large variations in DTW were observed with the same level of shoot biomass (Figure 4). For example, azuki bean (*V. angularis* var. *angularis*, ID3) and wild cowpea (*V. unguiculata* subsp. *dekindtiana*, ID64) have similar shoot biomass in the 30-cm pipe (8.8 g per plant and 8.2 g per plant, respectively) but the DTW was quite different (7.2 and 19.1 days, respectively). Another example is *V. vexillata* var. *vexillata* (ID51) and *V. vexillata* var. *angustifolia* (ID52). Their shoot dry weights in the 30-cm pipe were almost the same (6.3 g per plant in both), but the DTW was 19.2 and 12.8 days, respectively. These examples indicate that there is some possibility to improve the DTW without decrease in shoot biomass.

### Relationship of drought tolerances between the pipe and pot experiments

Since the tolerant accessions were not same between the pipe and the pot experiments, it can be concluded that different mechanisms were required. In the pipes, water loss was fast in the upper soil layers, but it was continuously replenished from the bottom. Thus, in this condition, the root characteristics were important to maintain shoot growth. In the pots, however, water was rapidly lost from the soil, and roots could hardly contribute to maintain the water uptake. As such, our results demonstrated three types of responses against drought in the genus *Vigna*.

The first type of response was observed in the accessions with low RSW and long DTW. Examples include *V. aconitifolia* (ID39), *V. subterranea* (ID69), and *V. unguiculata* (ID62) in Group IV (Figure 4). These accessions might be isohydric (McDowell et al., 2008), with stomata closing rapidly after the leaf water starts decreasing, to avoid further leaf dehydration. This type of plants are sometimes associated with severe growth inhibition even under mild drought stress, probably because the isohydric regulation is related to early loss of cell wall elasticity, which inhibits leaf expansion (Harb et al., 2010). On the other hand, the early stomatal closure helps prevent water loss from the leaf cells and might enable long survival under prolonged drought conditions. These accessions might be adapted to environments where severe drought stress frequently occurs.

The second type of response was observed in the accessions with high RSW and short DTW. Examples include *Vigna* sp. NI1135 (ID28 in Group I), *V. radiata* var. *sublobata* (ID27 in Group II), and *V. minima* (ID19, 20 in Group III). These accessions might be anisohydric (Skelton et al., 2015), maintaining high transpiration and photosynthetic rates under soil drying conditions, as seen in ID28 (Figure 9). This might help maintain shoot growth under conditions of relatively mild water deficiency. However, high water consumption makes the survival of plants difficult during prolonged soil water deficiency, which could be lethal. These accessions might have an advantage in environments where mild drought stress occurs intermittently but the severe ones rarely occur.

The third pattern is observed in the accessions with high RSW and long DTW. Examples are *V. vexillata* var. *ovata* (ID55 in Group I), wild accessions of *V. aconitifolia* (ID40–42 in Group II), and *V. vexillata* var. *lobatifolia* (ID59 in Group III). Although key tolerance mechanisms in both pipe and pot experiments could not be identified, these accessions seem to be interesting genetic resources for further studies to unveil the tolerance mechanisms.

## Conclusion

We identified 19 *Vigna* accessions as tolerant based on RSW of non-terminal drought in the vegetative stage in the pipe experiment. These accessions showed higher RSW than that in the domesticated accessions, which are widely cultivated in drought-prone regions (i.e., cowpea, bambara groundnut, moth bean, and black gram). Large differences in tolerance were detected in genetically close accessions, implying that genes determining tolerance can be identified through genetic approaches, such as linkage analysis, following bi-parental crossing. Accessions with high RSW showed good plant water status which might be due to small shoot biomass (small water consumption). Other plant traits such as deep rooting and increase in the root biomass were appeared as important tolerance-related factors, but the variation could not be explained by simple tolerance factors alone. Moreover, a large variation was observed in the pot experiment of terminal drought. However, the accessions with long DTW were different from the accessions with high RSW in the pipe experiment, suggesting that different tolerance mechanisms are needed for non-terminal and terminal drought conditions. It should be noted that some accessions showed tolerance for both drought conditions, although further studies are necessary for clarifying the tolerance mechanisms. As such, *Vigna* accessions can be considered good genetic resources for developing drought-adaptive crops and exploring the adaptation of this genus against drought-prone environments. Further, the genomes of some domesticated species have been already sequenced, including that of *V. unguiculata* (Muñoz-Amatriaín et al., 2017), *V. radiata* (Kang et al., 2014), and *V. angularis* (Kang et al., 2015; Sakai et al., 2015). This information strongly suggests the use of *Vigna* accessions for studies of stress tolerance.

## Author contributions

KI, NT, and KN: conception and design of the work; KI, YT, and CM: data acquisition; KI, YT, CM, NT, and KN: data analysis and interpretation; KI, NT, and KN: manuscript writing and revision.

### Conflict of interest statement

The authors declare that the research was conducted in the absence of any commercial or financial relationships that could be construed as a potential conflict of interest.
