# Supplementary material for: Diversity of Drought Tolerance in the Genus Vigna
Source: Front Plant Sci. 2018 Jun 15;9:729. doi: 10.3389/fpls.2018.00729 (PMC6014140; doi:10.3389/fpls.2018.00729)
Supplement: Supplementary file 1 [file Data_Sheet_1.DOCX]

Supplementary Material

**Diversity and evolution of drought tolerance in the genus *Vigna***

**Kohtaro Iseki*, Yu Takahashi, Chiaki Muto, Ken Naito, Norihiko Tomooka**

*** Correspondence:** Kohtaro Iseki: iseki83@affrc.go.jp

## Supplementary Figure S1


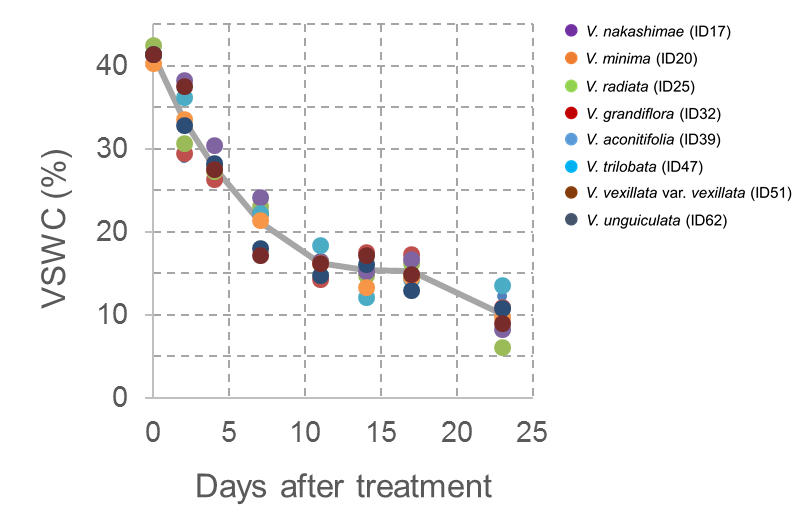


## Supplementary Figure S1. Volumetric soil water content in the pot experiment. Time-course changes in the volumetric soil water content (VSWC) measured at midday during the drought treatment. Each data point is the value for one pot (planted with one accession), and data of eight pots are shown at each time point. The lines represent the averages of the eight pots.
