# Supplementary material for: Diversity of Drought Tolerance in the Genus Vigna
Source: Front Plant Sci. 2018 Jun 15;9:729. doi: 10.3389/fpls.2018.00729 (PMC6014140; doi:10.3389/fpls.2018.00729)
Supplement: Supplementary file 2 [file Data_Sheet_2.DOCX]

Supplementary Material

**Diversity and evolution of drought tolerance in the genus *Vigna***

**Kohtaro Iseki, Yu Takahashi, Chiaki Muto, Ken Naito, Norihiko Tomooka***

*** Correspondence:** Kohtaro Iseki: iseki83@affrc.go.jp

**Supplementary Figure S2**


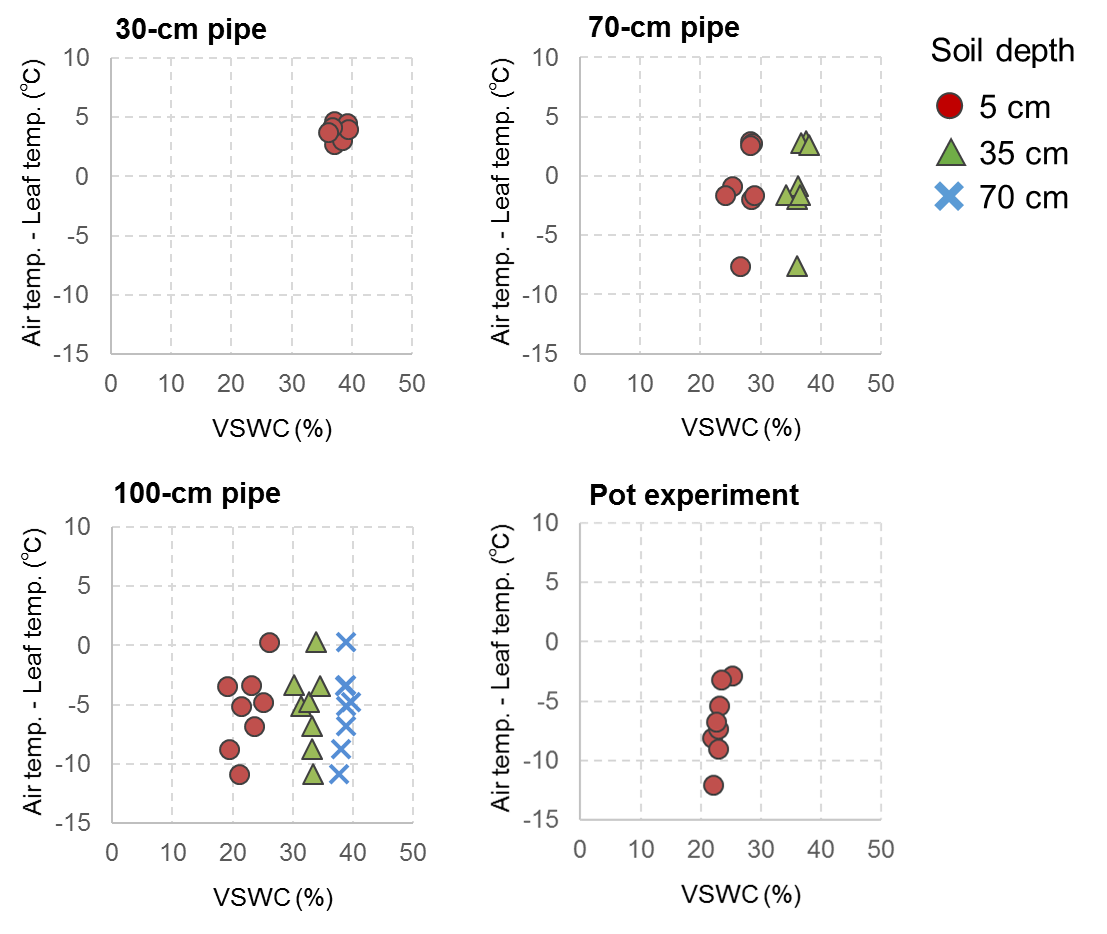


**Supplementary Figure S2.** Relationship between volumetric soil water content (VSWC) and air-leaf temperature difference in the pipe experiment and pot experiment. Data of the eight accessions shown in Figure 2 were used. VSWC are averages during from 14 to 25 days after treatment when large variation was observed among the different pipe height and pot experiment. Different symbols represent different soil depth for each accession.
