# Supplementary material for: Diversity of Drought Tolerance in the Genus Vigna
Source: Front Plant Sci. 2018 Jun 15;9:729. doi: 10.3389/fpls.2018.00729 (PMC6014140; doi:10.3389/fpls.2018.00729)
Supplement: Supplementary file 3 [file Data_Sheet_3.DOCX]

Supplementary Material

**Diversity and evolution of drought tolerance in the genus *Vigna***

**Kohtaro Iseki, Yu Takahashi, Chiaki Muto, Ken Naito, Norihiko Tomooka***

*** Correspondence:** Kohtaro Iseki: iseki83@affrc.go.jp

##
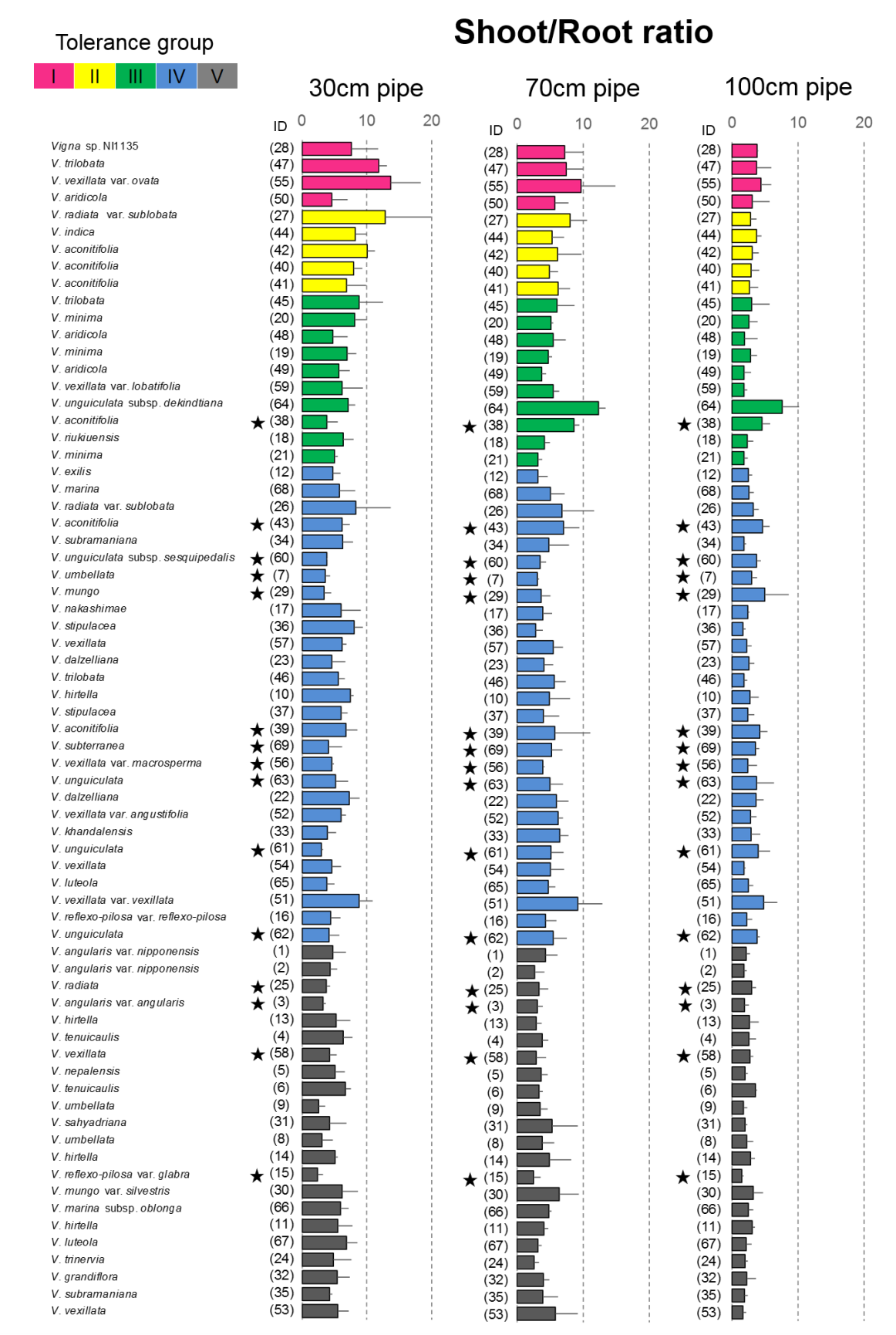
Supplementary Figure S3

**Supplementary Figure S3.** Shoot-root ratios for the 69 accessions in the pipe experiment. Data are the averages ± standard errors for the three trials. The five groups identified by cluster analysis are indicated using different colors, same as shown in Figure 4. Stars indicate domesticated accessions.
