# Supplementary material for: Diversity of Drought Tolerance in the Genus Vigna
Source: Front Plant Sci. 2018 Jun 15;9:729. doi: 10.3389/fpls.2018.00729 (PMC6014140; doi:10.3389/fpls.2018.00729)
Supplement: Supplementary file 4 [file Data_Sheet_4.DOCX]

Supplementary Material

**Diversity and evolution of drought tolerance in the genus *Vigna***

**Kohtaro Iseki*, Yu Takahashi, Chiaki Muto, Ken Naito, Norihiko Tomooka**

*** Correspondence:** Kohtaro Iseki: iseki83@affrc.go.jp

## Supplementary Figure S4


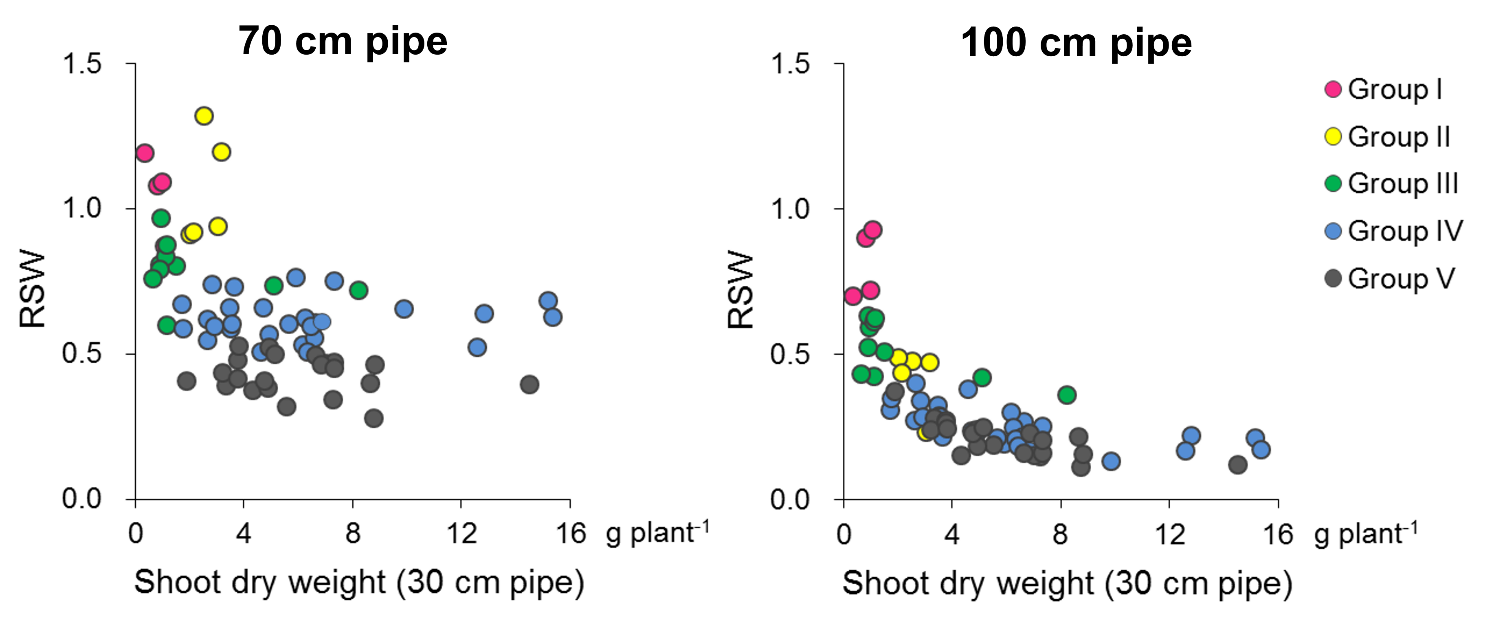


**Supplementary Figure S4.** Relationship between shoot dry weight in the 30-cm pipes and the relative shoot dry weight (RSW) in the 70-cm and 100-cm pipes. Data are the averages of three trials. The five groups identified by cluster analysis are indicated using different colors, same as shown in Figure 4.
