# Supplementary material for: Diversity of Drought Tolerance in the Genus Vigna
Source: Front Plant Sci. 2018 Jun 15;9:729. doi: 10.3389/fpls.2018.00729 (PMC6014140; doi:10.3389/fpls.2018.00729)
Supplement: Supplementary file 5 [file Table_1.DOCX]

**Table S1** Experimental date and meteorological conditions during drought treatments. Temperature, vapor pressure deficit and radiation are the mean ± SD during the experiment. Daytime data and night data are the averages during the time from 6 to18 and 18-6, respectively.

|  | Trial 1 (2013) | Trial 2 (2013) | Trial 3 (2014) |
| --- | --- | --- | --- |
| Sowing date | 27 June | 5 September | 22 April |
| Drought onset date | 18 July | 26 September | 13 May |
| Sampling date | 7 August | 18 October | 4 June |
| Daytime temperature (°C) | 32.4 ± 3.5 | 28.3 ± 3.0 | 29.5 ± 3.4 |
| Daytime vapor pressure deficit (hPa) | 17.7 ± 6.8 | 12.5 ± 5.8 | 17.4 ± 6.4 |
| Night temperature (°C) | 23.7 ± 2.3 | 22.4 ± 2.0 | 20.6 ± 1.8 |
| Night vapor pressure deficit (hPa) | 2.3 ± 1.2 | 1.2 ± 0.9 | 5.0 ± 2.5 |
| Radiation (MJ day^-1^) | 18.5 ± 6.1 | 13.5 ± 5.9 | 14.2 ± 5.3 |
